# Editorial: Artificial intelligence in nephrology

**DOI:** 10.3389/fneph.2023.1270769

**Published:** 2023-09-06

**Authors:** Francesco Bellocchio, Hanjie Zhang

**Affiliations:** ^1^ Clinical Advanced Analytics - International Data Science, Fresenius Medical Care, Bad Homburg, Germany; ^2^ Computational Statistics & Artificial Intelligence, Renal Research Institute, New York, NY, United States

**Keywords:** artificial intelligence, hemodialysis, machine learning, chronic kidney disease, support decision systems

Artificial Intelligence (AI) has emerged as a revolutionary technology with vast potential to transform various sectors, and healthcare is no exception. In the realm of nephrology, AI presents unprecedented opportunities to enhance patient care, improve diagnostics, streamline workflows, and facilitate more precise treatment decisions. The Research Topic of *Artificial Intelligence in Nephrology* aims to explore the transformative impact of AI on nephrology, discussing its applications in diagnosis, risk prediction, treatment optimization, and patient monitoring. While acknowledging the challenges and ethical considerations associated with AI implementation, this Research Topic emphasizes the immense potential of AI in revolutionizing nephrology and paving the way for a more efficient and personalized approach to patients with kidney diseases.

The five papers published in this Research Topic provided a glimpse of the many ways in which AI is being used in nephrology today.

A first important aspect covered by the papers regards the use of AI for enhancing diagnosis and prognosis. Both these medical tasks are crucial for effective management and treatment. AI algorithms can assist nephrologists in making timely and accurate diagnoses and prognosis, ultimately leading to improved patient outcomes. Machine learning models can analyze vast amounts of patient data, including laboratory results, medical imaging, and clinical notes, to identify patterns and detect anomalies that may indicate kidney related disorders. These algorithms can aid in the early detection of conditions such as chronic kidney disease (CKD), arteriovenous fistula problems, and cardiovascular complications.

The first paper presents a prognosis tool for cardiovascular events for non-dialysis-dependent CKD (Neri et al.). This tool has the potential to improve the identification of patients at high risk for cardiovascular events and could lead to earlier intervention and better outcomes. For the diagnosis task, the second paper discusses the use of AI to automate the diagnosis of kidney diseases from kidney biopsies (Basso et al.). This approach could help to reduce the time and cost of kidney biopsy interpretation and could also improve the accuracy of diagnosis.

The prediction ability of AI solutions, created on large amount of data, can be leveraged also in critical and unexpected situations like coronavirus disease 2019 (COVID-19) pandemic. The fifth paper is an example of such application (Duan et al.). It describes the development of a machine learning model to predict SARS-CoV-2 infection in dialysis patients. Considering the higher impact of COVID-19 among this fragile population, this model could be used to identify patients at high risk for COVID-19 and could help to prevent the spread of the virus within dialysis clinics.

In general, these models can integrate patient-specific data, including demographics, medical history, genetics, and lifestyle factors, to generate personalized risk scores and treatment recommendations. The personalization of the risk scores can aid in optimizing treatment protocols and devoting healthcare resources where more needed. Furthermore, by analyzing patient characteristics, response to treatment, and data from clinical trials, AI algorithms can assist in identifying the most effective interventions and tailoring treatment plans to individual patients. This personalized approach can improve therapeutic outcomes, minimize adverse events, and enhance patient satisfaction.

Another class of tools to facilitate personalized healthcare is represented by AI-powered monitoring systems. They offer real-time insights into patients’ overall health. Continuous monitoring of vital signs, laboratory results, and wearable device data can provide a comprehensive overview of a patient’s condition, allowing for early detection of complications or changes that require intervention. These AI-driven monitoring systems can significantly improve patient care by enabling timely adjustments to treatment plans and reducing the risk of adverse events. The fourth paper explores specifically this topic (Canaud et al.). It discusses the importance of closely monitor CVD in CKD5D patients and intervene early. Recently, home-used, self-operated, connected medical devices have emerged, providing convenient and automated monitoring during the interdialytic period. These sensor devices, equipped with Wi Fi or Bluetooth, analyze data and upload results to secure servers. Patients can access data through smartphone applications, while health care professionals can view it through web interfaces. The use of wearable sensors offers a multidimensional assessment of CKD5D patients, enabling the detection of unexpected disorders and aiding physicians in evaluating treatment responses. As the technology becomes more accessible and affordable, it has the potential to enhance personalized CKD5D patient management, ultimately improving their quality of life and survival.

AI methods can also be exploited to investigate the risk factors of specific disease and acute event. This is the case of the study of the third paper (Nong et al.). The aim of the study was to identify risk factors for severe acute kidney injury (AKI) in patients with acute myocardial infarction (AMI). The researchers analyzed data from 2022 patients in the Medical Information Mart for Intensive Care. Patients with severe AKI had higher in-hospital mortality and longer intensive care stays compared to those without AKI. The study found that several factors independently increased the risk of severe AKI in patients with AMI. These findings highlight the importance of monitoring these factors to identify patients at higher risk for severe AKI and improve prognosis following AMI.

While the potential of AI in nephrology is immense, its implementation comes with challenges and ethical considerations. Privacy and data security are paramount, as AI relies on vast amounts of patient data. Ensuring appropriate data anonymization, consent, and adherence to regulatory frameworks is crucial to maintain patient trust and protect sensitive information.

Additionally, transparency and interpretability of AI algorithms are essential to build confidence among healthcare professionals. Nephrologists must understand how AI arrives at its recommendations to effectively incorporate them into clinical decision-making processes.

In conclusion, this Research Topic highlights five different examples how Artificial Intelligence holds transformative potential in nephrology, revolutionizing diagnosis, risk prediction, treatment optimization, and patient monitoring. By leveraging AI technologies, nephrologists can provide more accurate diagnoses, identify high-risk patients, optimize treatment plans, and enhance patient monitoring. However, challenges such as data privacy, algorithm transparency, and ethical considerations must be addressed to ensure the responsible and beneficial integration of AI in nephrology. Embracing the power of AI in nephrology can unlock new possibilities and lead to improved patient outcomes, ultimately shaping a future where kidney care is more efficient, personalized, and effective.

## Author contributions

FB: Writing – original draft, Writing – review & editing. HZ: Writing – original draft, Writing – review & editing.

